# Fresh human cardiac tissue for translational research: A novel method of sampling deceased organ donors

**DOI:** 10.1016/j.xjtc.2023.03.020

**Published:** 2023-04-17

**Authors:** Varun Sharma, James A.L. Grant, Shivanand Gangahanumiah, Aashima Singh, Claire L. Gordon, Fiona James, Rohit D'Costa, Graham Starkey, Jaishankar Raman

**Affiliations:** a Department of Surgery, Austin Health, Melbourne Medical School, The University of Melbourne, Melbourne, Victoria, Australia; b Brian F. Buxton Department of Cardiac and Thoracic Aortic Surgery, Austin Health, Heidelberg, Melbourne, Victoria, Australia; c Department of Medicine, Melbourne Medical School, The University of Melbourne, Melbourne, Victoria, Australia; d Department of Microbiology and Immunology, Peter Doherty Institute for Infection and Immunity, The University of Melbourne, Melbourne, Victoria, Australia; e Department of Infectious Diseases, Austin Health, Heidelberg, Melbourne, Victoria, Australia; f Department of Intensive Care Medicine, Melbourne Health, Melbourne, Victoria, Australia; g Donatelife Victoria, Carlton, Victoria, Australia; h Liver Transplant Unit, Austin Health, Heidelberg, Melbourne, Victoria, Australia; i Department of Cardiac Surgery, St Vincent's Hospital, Melbourne, Victoria, Australia

**Figure:**
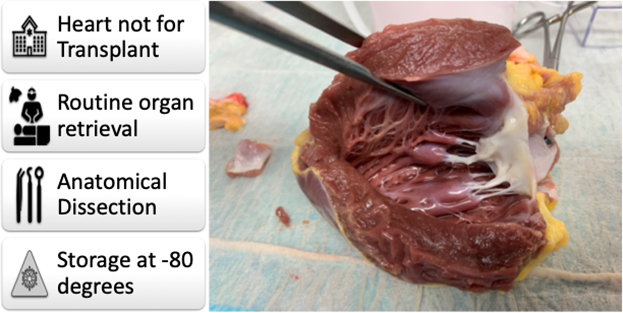
Protocol for collecting cardiac tissue from donors where the heart is not for transplant.

**Central Message**

We describe novel sampling of fresh human cardiac tissue for use in research from deceased organ donors where the heart was not suitable for transplantation.

A major barrier to translating basic cardiovascular findings to clinical practice is limited access to fresh human cardiac tissue. The heart is rarely excised, and intraoperative sampling carries life-threatening risk and is resource-intensive. Translational cardiac research is therefore often reliant on animal models, which correlate poorly with human disease.^1^ Only 30% of deceased organ donors have hearts suitable for organ transplantation,^2^ providing an opportunity for donation of the heart for use in research. The Australian Donation and Transplantation Biobank (ADTB) facilitates the donation of tissue samples for use in research using a centralized system integrated into the deceased organ donation program (Reference Transplant Direct paper^3^). We describe the technical aspects of collecting, processing, and storing fresh human cardiac tissue for use in future research as part of ADTB.

## Ethics and Consent

Ethics approval for ADTB has been described previously.^3^ Approval for this project was obtained on June 21, 2021 (HREC/73660/Austin-2021).

## Heart Procurement

**Figure 1:**
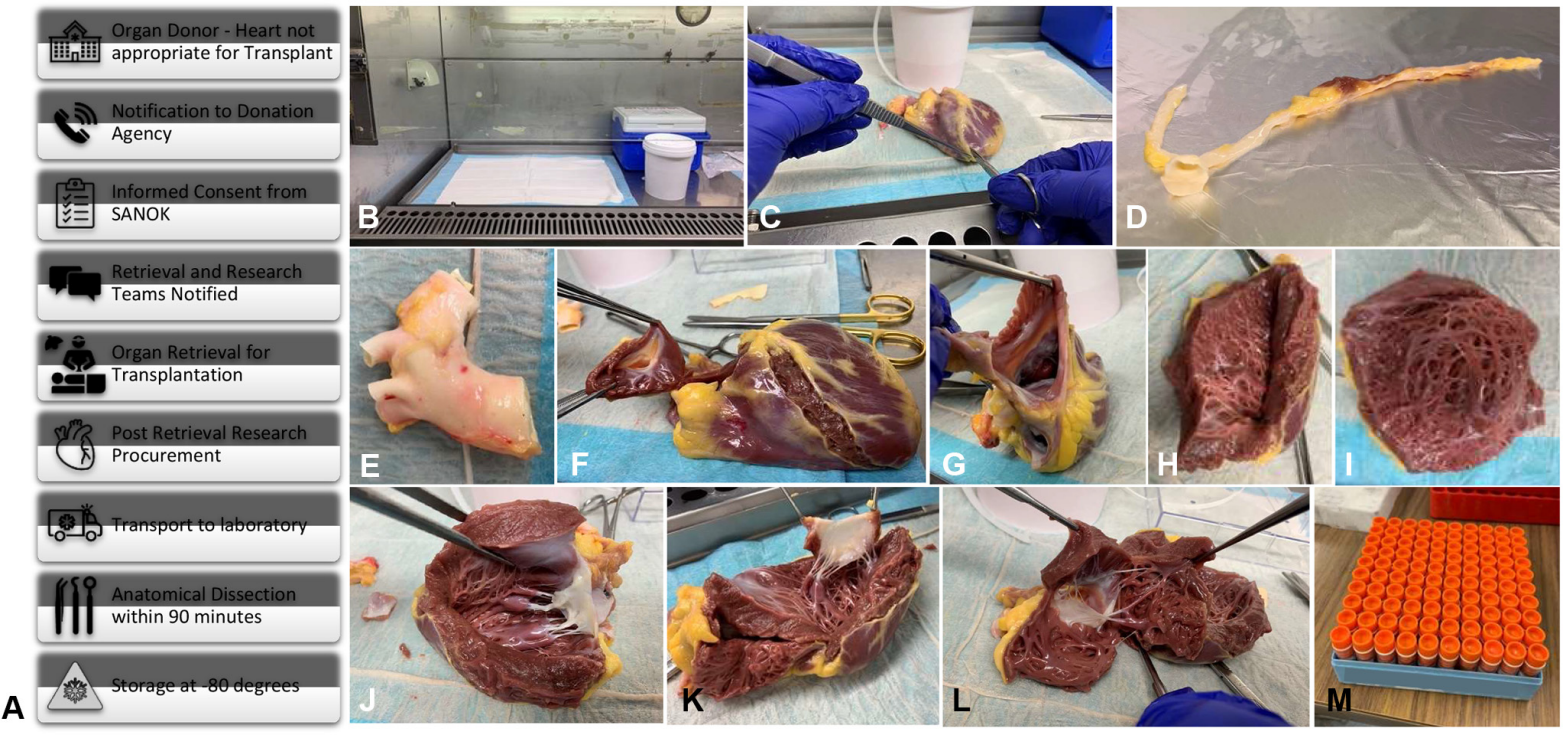
Protocol for collecting fresh cardiac tissue from organ donors where the heart is not used for transplant. A, The workflow. B, Setup under laminar hood. C, Dissection of the coronary arteries to the level of the aorta (D), after which the aorta is excised with aortic root (E), followed by excision of the pulmonary valve (F). The atria are opened through the appendages (G), and ventricles opened through incisions parallel to the interventricular septum (H) to obtain myocardial tissue (I). Further incisions into ventricular chambers (J) allows dissection of mitral (K) and tricuspid valves (L) with papillary muscles and trabecular attachments. M, When not being stored whole, these tissues can be stored in 2 mL Eppendorf tubes. *SANOK*, Senior available next of kin.

The donation coordination agency obtains informed consent for donation to the ADTB from the senior available next of kin.^3^ If the heart is not suitable for transplantation (Figure 1, *A*), the heart is excised immediately after all organs for transplantation have been retrieved to minimize heart ischemic time. Access is via a median sternotomy, after which the pericardium is opened and aorta transected in the descending aorta with 2 to 3 cm of the arch vessels. The vena cavae and trachea are ligated, and heart and lungs explanted en-bloc. In cases where lungs are being retrieved for transplantation, unused cardiac tissue is dissected out on the back table. Samples are stored on ice and transported to the laboratory (Figure 1, *A*).

## Tissue Processing

We dissect the coronary arteries, atrium, ventricles, interventricular septum, atrioventricular valves, and semilunar valves as follows.

• The specimen is placed under a laminar flow hood, onto an absorbent under pad (Figure 1, *B*).
• The left and right coronary artery systems are excised along the epicardial fat and myocardium (Figure 1, *C*) along the interventricular atrioventricular grooves (Figure 1, *C*) to the level of the aorta (Figure 1, *D*).
• The aorta is separated with circumferential dissection along the aortic root (Figure 1, *E*) with the aortic valve through a 5-mm incision into the left ventricular outflow tract.
• The pulmonary artery and pulmonary valve are separated with circumferential dissection via the right ventricular outflow tract (Figure 1, *F*).
• Atrial tissue is obtained by entering the right and left atrial appendages. For the right atrium, the incision is extended to level of the vena cavae (see Figure 1, *G*).
• The right ventricle is opened from the right ventricle 1-cm parallel along the posterior and anterior interventricular septum (Figure 1, *H*) to obtain samples of the right ventricle (Figure 1, I).
• The mitral and tricuspid valves are dissected by further opening the left (Figure 1, *J* and *K*) and right (Figure 1, *L*) ventricles, and excised with their trabecular and papillary muscular attachments.
• The previous incisions are continued along their respective atrioventricular septa, partitioning the specimen into independent atria and ventricle chambers (Figure 1, *L*).
• For select projects, samples are further dissected into 1-cm^3^ blocks and placed into labeled 2-mL screw cap cryovials (Eppendorf tubes) (Figure 1, *M*).

All samples were processed within 90 minutes of arriving at the laboratory. Approximately 100 tissue blocks can be stored per heart (Figure 1, *M*).

## Tissue Storage

For anatomical dissection specimens, tissues are wrapped in 3 layers of aluminium foil and stored in polyethylene boxes in −80 °C freezers with a 24-hour alarm. For laboratory dissection, the tubes are stored by rapid cryopreservation by first submerged in liquid nitrogen (−196 °C) for snap freezing (<5 minutes) and then transferred to −80 °C freezers for long-term storage. Specimens catalogued using Redcap (Research Electronic Data Capture) and FreezerPro (Brooks Life Sciences) databases, labeled with details, including unique ADTB study number, cardiac region, slot number, and box position.

## Discussion

We describe a new process to obtain fresh frozen cardiac tissue for use in translational research donated by deceased organ donors, with minimal disruption to clinical workflow and outlay of resources. Between April 2020 and October 2022, 31 hearts were donated to ADTB with a median age of 52 years, with 48% women. Samples have been used for immunohistochemical studies, biochemical analysis, mass spectrometry, cell culture, and novel translational technologies such as spectrophotometric analysis. The preservation of cellular detail, tissue lipids, metabolic features, and proteins makes this approach feasible.^4^^,^^5^

## References

[1] Zhu Y., Jackson D., Hunter B., Beattie L., Turner L., Hambly B. (2022). Models of cardiovascular surgery biobanking to facilitate translational research and precision medicine. ESC Heart Fail.

[2] Transplant Sciety of Australia and New Zealand (2022). Clinical guidelines for organ transplantation from deceased donors. Version 1.10. https://tsanz.com.au/storage/documents/TSANZ_Clinical_Guidelines_Version-110_Final.pdf.

[3] Sharma V.J., Starkey G., D'Costa R., James F., Mouhtouris E., Davis L. (2023). Australian donation and transplantation Biobank: a research Biobank integrated within a deceased organ and tissue donation program. Transplant Direct.

[4] Stock A.T., Parsons S., Sharma V.J., James F., Starkey G., D'Costa R. (2022). Intimal macrophages develop from circulating monocytes during vasculitis. Clin Transl Immunology.

[5] Stock A.T., Parsons S., D'Silva D.B., Hansen J., Sharma V., James F. (2023). Mechanistic target of rapamycin inhibition prevents coronary artery remodeling in a murine model of Kawasaki disease. Arthritis Rheumatol.

